# SuperResNET: Model‐Free Single‐Molecule Network Analysis Software Achieves Molecular Resolution of Nup96

**DOI:** 10.1002/aisy.202400521

**Published:** 2024-12-25

**Authors:** Yahongyang Lydia Li, Ismail M. Khater, Christian Hallgrimson, Ben Cardoen, Timothy H. Wong, Ghassan Hamarneh, Ivan R. Nabi

**Affiliations:** ^1^ Department of Cellular and Physiological Sciences, Life Sciences Institute University of British Columbia Vancouver BC V6T 1Z3 Canada; ^2^ School of Computing Science Simon Fraser University Burnaby BC V5A 1S6 Canada; ^3^ Department of Electrical and Computer Engineering, Faculty of Engineering and Technology Birzeit University Birzeit P627 Palestine; ^4^ School of Biomedical Engineering University of British Columbia Vancouver BC V6T 1Z3 Canada

**Keywords:** direct stochastic optical reconstruction microscopy, machine learning, network analysis, nuclear pores, Nup96, SuperResNET software

## Abstract

SuperResNET is an integrated machine learning‐based analysis software for visualizing and quantifying 3D point cloud data acquired by single‐molecule localization microscopy (SMLM). SuperResNET computational modules include correction for multiple blinking of single fluorophores, denoising, segmentation (clustering), feature extraction used for cluster group identification, modularity analysis, blob retrieval, and visualization in 2D and 3D. Here, a graphical user interface version of SuperResNET was applied to publicly available direct stochastic optical reconstruction microscopy (dSTORM) data of nucleoporin Nup96 and Nup107 labeled nuclear pores that present a highly organized octagon structure of eight corners. SuperResNET effectively segments nuclear pores and Nup96 corners based on differential proximity threshold analysis from 2D and 3D SMLM datasets. SuperResNET quantitatively analyzes features from segmented nuclear pores, including complete structures with eightfold symmetry, and from segmented corners. SuperResNET modularity analysis of segmented corners from 2D SMLM distinguishes two modules at 10.7 ± 0.1 nm distance, corresponding to two individual Nup96 molecules. SuperResNET is therefore a model‐free tool that can reconstruct network architecture and molecular distribution of subcellular structures without the bias of a specified prior model, attaining molecular resolution from dSTORM data. SuperResNET provides flexibility to report on structural diversity in situ within the cell, providing opportunities for biological discovery.

## Graphical abstract

SuperResNET is a powerful integrated software that reconstructs network architecture and molecular distribution of subcellular structures from single molecule localization microscopy datasets. SuperResNET segments the nuclear pore complex and corners, extracts size, shape, and network features of all segmented nuclear pores and uses modularity analysis to identify two Nup96 molecules within nuclear pore corners.

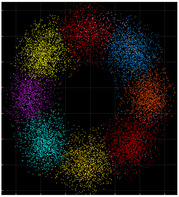

## 1 Introduction

Single‐molecule localization microscopy (SMLM) is a super‐resolution microscopy technique that generates 2D or 3D point cloud data by localizing target proteins labeled by isolated fluorophores, providing lateral resolution of 5–20 nm and axial resolution of 10–30 nm using photoactivated localization microscopy,^[^ ^1^, ^2^ ^]^ stochastic optical reconstruction microscopy (STORM),^[^ ^3^, ^4^ ^]^ and point accumulation for imaging in nanoscale topography (PAINT).^[^ ^5^, ^6^ ^]^ MINFLUX nanoscopy, which also generates point cloud data by localizing single fluorophores labeling the protein of interest, achieved 1–3 nm 3D resolution.^[^ ^7^, ^8^ ^]^ More recently, resolution enhancement by sequential imaging, implemented using DNA‐PAINT, further improved resolution to Angstrom level.^[^ ^9^ ^]^ SMLM has been applied to study biological structures including actin, microtubules, nuclear pore complex, clathrin‐coated pits, caveolae, and focal adhesions,^[^ ^10^, ^11^ ^]^ providing visualization and reconstruction at the nanometer scale.

While software packages for reconstruction and visualization of SMLM data are well‐developed,^[^ ^12^ ^]^ approaches for quantitative analysis of 2D or 3D point cloud data remain limited. Clustering analysis methods for SMLM include statistical, Bayesian, density‐based, correlation‐based, tessellation‐based, image‐based, and machine learning‐based approaches.^[^ ^13^, ^14^ ^]^ SuperResNET is a machine learning based‐approach where networks are constructed from point cloud SMLM data and used to understand molecular architecture of biological structures.^[^ ^15^ ^]^ Point clouds consist of distributions of nodes within 2D or 3D space that can form a network by construction of edges between nodes within a defined proximity threshold. Network analysis comprises the study of how nodes interact within this network, comprising size, shape, and network features, providing information on the underlying organization and architecture of the network. A more extensive description of the application of network, or graph, analysis to SMLM data can be found here.^[^ ^14^ ^]^

Previously, we applied SuperResNET network analysis to SMLM data for caveolin‐1 (CAV1) and identified caveolae and three distinct classes of noncaveolar scaffolds.^[^ ^16^, ^17^, ^18^ ^]^ Modularity analysis showed that small CAV1 scaffolds combine to form larger scaffolds and caveolae,^[^ ^17^ ^]^ and convex hull analysis detected changes in caveolae structure due to point mutation in the caveolin scaffolding domain.^[^ ^19^ ^]^ Here, we present a user‐friendly graphical user interface (GUI) version of SuperResNET for 2D or 3D SMLM network analysis,^[^ ^15^, ^17^ ^]^ providing fast cluster identification and classification from SMLM point cloud datasets. SuperResNET GUI is robust for denoising SMLM data, segmenting clusters, extracting features of clusters, and using machine learning to classify clusters. Additionally, SuperResNET GUI enables visualization of segmented clusters and retrieves clusters based on selected feature(s).

The nuclear pore complex is used extensively as a reference structure for super‐resolution microscopy because of its well‐defined structures and abundance in the cell.^[^ ^20^ ^]^ About 30 different nucleoporins are found in the nuclear pore complex, and electron microscopy has identified the structures of most of the nucleoporins (Figure S1, Supporting Information).^[^ ^21^ ^]^ Here, we evaluate the capability of SuperResNET GUI to analyze publicly available 2D dSTORM data for nucleoporin Nup96^[^ ^20^ ^]^ and demonstrate its ability to effectively segmenting nuclear pores and Nup96 corners. While most nuclear pores are incomplete, SuperResNET GUI is able to extract the size, shape, and network features of all segmented blobs and selected complete ones. Modularity analysis of segmented corners distinguishes 2 modules at 10.7 ± 0.1 nm distance corresponding to the 2 individual Nup96 molecules, known to be located at 12 nm distance within the nucleopore corners.^[^ ^21^ ^]^ SuperResNET network analysis of dSTORM data of Nup96,^[^ ^20^ ^]^ with a predicted resolution of 13.3 ± 0.1 nm, therefore resolves molecules at 12 nm distance. SuperResNET applied to 3D PAINT data for Nup107 reconstructed by particle fusion^[^ ^22^ ^]^ also effectively segmented Nup107 corners.

## 2 Results

### 2.1 SuperResNET GUI

**Figure 1:**
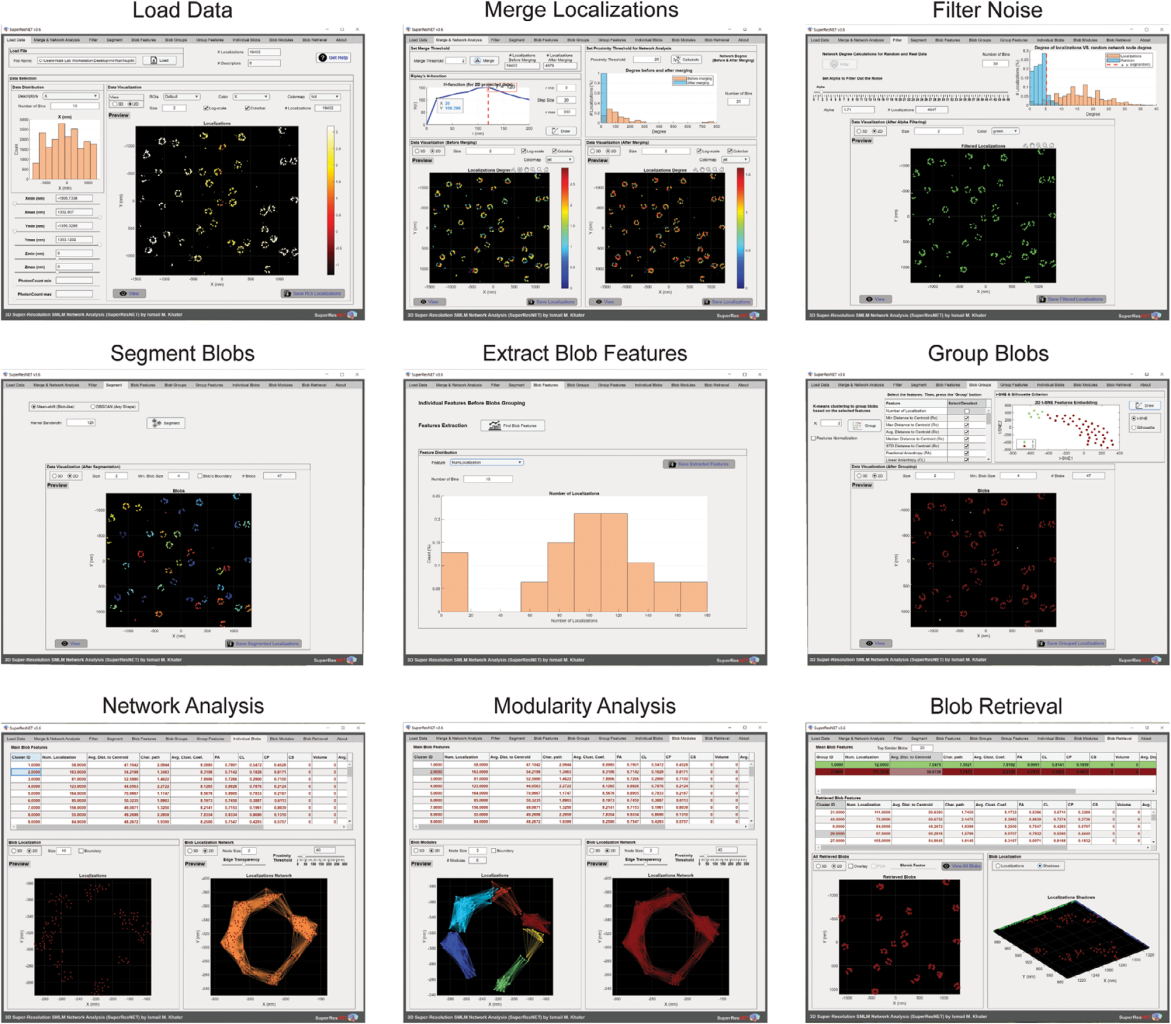
Overview of SuperResNET software. SuperResNET GUI is an integrated software to quantify and visualize 2D/3D point cloud data. The pipeline of SuperResNET includes loading point cloud data, correcting/merging of multiple blinks originating from the same fluorophore, filtering of background noise, segmentation of blobs, extraction of blob features, grouping based on blob features, network and modularity analysis of individual blobs, retrieval of representative blobs as well as 2D and 3D visualization.

SuperResNET is an integrated software to visualize and quantify 3D point cloud data acquired by SMLM. The GUI provides users an easy access to loading data, correction of multiple blinks originating from the same fluorophore, filtering of background noise, segmentation of clusters, quantification of cluster features, grouping based on cluster features, and network and modularity analysis of individual clusters as well as 2D and 3D visualization under GUI tabs (**Figure** **1**). Key features of SuperResNET include: *Load data*: loads point cloud data and provides histogram visualization of localization and the associated metadata; *Merge & Network analysis*: removes artifacts from multiple blinks by iteratively merging localizations within a merge threshold, and assesses scale of clustering by Ripley's H‐function; *Filter*: denoises data based on comparison with degree distribution of a random network; *Segment*: provides mean‐shift segmentation for blob‐like structures and DBSCAN to segment structures with other shapes; *Blob features*: extraction, quantification, and visualization of 30 features comprising size, shape, topology/hollowness, statistical and network features of segmented blobs and reports histograms of the features; *Group*: assigns group to segmented blobs based on selected features through K‐means clustering and reports color‐coded histograms of grouped features, and assesses grouping using t‐SNE or silhouette methods; *Individual blobs*: visualizes individual blobs with network connection at user‐defined proximity thresholds; *Blob modules*: extracts and visualizes modules of interacting molecules at user‐defined proximity thresholds for individual blobs; *Blob retrieval*: retrieves representative blobs from each group with visualization of localization, network, and boundaries in 2D and 3D. **Table** **1** provides guidelines for users to set parameters. A more detailed description can be accessed at https://www.medicalimageanalysis.com/software/superresnet/documentation or from the Get Help button under the Load data tab in SuperResNET GUI.

**Table 1:** Guidelines for setting SuperResNET parameters.

GUI tab	Parameter	Objective	How to set parameters		Limitations
			Set:	Guidelines	
Merge & Network Analysis	Merge threshold	Combines localizations within the merge threshold by iterative merging replacing them with an estimated average location	To zero	If multiple blinks are removed using other approaches (e.g., when localizations are persistent over n frames)	SuperResNET merge is best applied to datasets with saturated number of localizations
			Based on localization precision/resolution of the imaging system	Resolution of the imaging system depends on the labeling approach used (i.e., size/number of antibodies)	
			Based on biological ground truth	i.e., 144 ± 39 CAV1's per caveolae was used to define merge thresholds^[^ ^15^, ^32^ ^]^	
Merge & Network Analysis	Proximity threshold	Defines maximum distance between localizations for network construction and calculating node degree. Used for filtering and for determining blob features.	Based on cluster size	Determined using Ripley's H‐function (see Figure 2B)	Can cause small clusters to be removed during denoising, if set too high
			Estimate based on prior knowledge	i.e., known size of biological structure considering localization precision and type of antibody labeling	
			By parameter sweep to best distinguish two datasets	i.e., comparison of CAV1 blobs’ features that best differentiated cells expressing or not caveolae^[^ ^15^ ^]^	
Filter	Alpha	Denoises by removing localizations that are not clustered within user defined proximity threshold compared to randomized distribution	By comparing the degree of selected image and a randomized network	Localizations in the ROI are randomized and the average degree is calculated for actual and randomized network. Alpha is chosen to remove random localizations with degree lower than alpha × average	Where cell covers majority of ROI and has high localization density, the degree of actual and randomized network might not be well‐separated.
			By determining degree of localizations in a noncell background area.	Alpha is set to remove localizations with degree lower than background	
Segment	Mean shift kernel bandwidth	Mean shift algorithm is suitable for segmenting blob‐like clusters Kernel bandwidth is the expected standard deviation of the kernels used to fit the points.	Based on Ripley's H‐function	i.e., kernel bandwidth of 118 (for nuclear pores) and 12 (for corners) based on Ripley's H‐function (Figure 2B) for Nup96	High kernel bandwidth can over‐segment (fragment large structures) Low kernel bandwidth can under‐segment (combine near‐by structures)
			Based on prior biological knowledge		
			To optimize, apply various kernel bandwidths to the same ROI and compare segmentation to expected results		
	DBSCAN: MinPnts and Epsilon	DBSCAN: Suitable for clusters of all shapes. Clusters with localizations fewer than minimum number of points (MinPnts) are removed. Epsilon is the maximum distance between localizations within a cluster	Prior knowledge on biological structure can inform on MinPnt and epsilon	i.e., based on expected number of molecules or size of cluster.	High MinPnts can remove small clusters High Epsilon can over‐segment (fragment large structures) Low Epsilon can under‐segment (combine near‐by structures)
			By applying various combinations of MinPnts and Epsilon and comparing segmentation	i.e., combination of MinPnts and epsilon to best segment visually distinct pits and vesicles for clathrin^[^ ^23^ ^]^	
Group	K means	Classifies segmented blobs into one or multiple groups based on feature similarity	Number of groups can be determined from histogram of segmented features	i.e., histograms of various features show two peaks, suggesting the presence of two groups for nuclear pores (Figure S2, Supporting Information)	Feature distribution of blobs in different groups can overlap. Users need to specify number of groups. Potential to incorporate outside tools (multiple instance learning; X means) to determine number of groups.
			Based on prior biological knowledge		
			Using t‐SNE plot and silhouette in the Group tab quality		
Individual blobs	Proximity threshold	Defines maximum distance between localizations for network construction. Used for visualization and modularity analysis.	By applying a range of proximity thresholds and find the range that result in stable output (e.g., number of modules, distance between module centroids)	i.e., proximity thresholds 9–14 nm result in stable distance between module centroids for Nup96 corners (Figure 6); proximity thresholds 60–170 nm result in stable number of modules in caveolae and scaffolds for CAV1 dataset^[^ ^17^ ^]^	
Blob modules					
Blob retrieval	Shrink factor	A scalar parameter between 0 and 1 that defines how tight the 2D boundary or 3D triangulation is around localizations. 0 shrink gives the convex hull and 1 shrink gives the smallest single connected shape that includes all the points in the dataset.			Low shrink factor can exclude fine structural details. High shrink factor generally increases the irregularity of the structure and is more sensitive to noise.

### 2.2 SuperResNET Analysis of 2D DSTORM Data for Nup96‐Labeled Nuclear Pores

**Figure 2:**
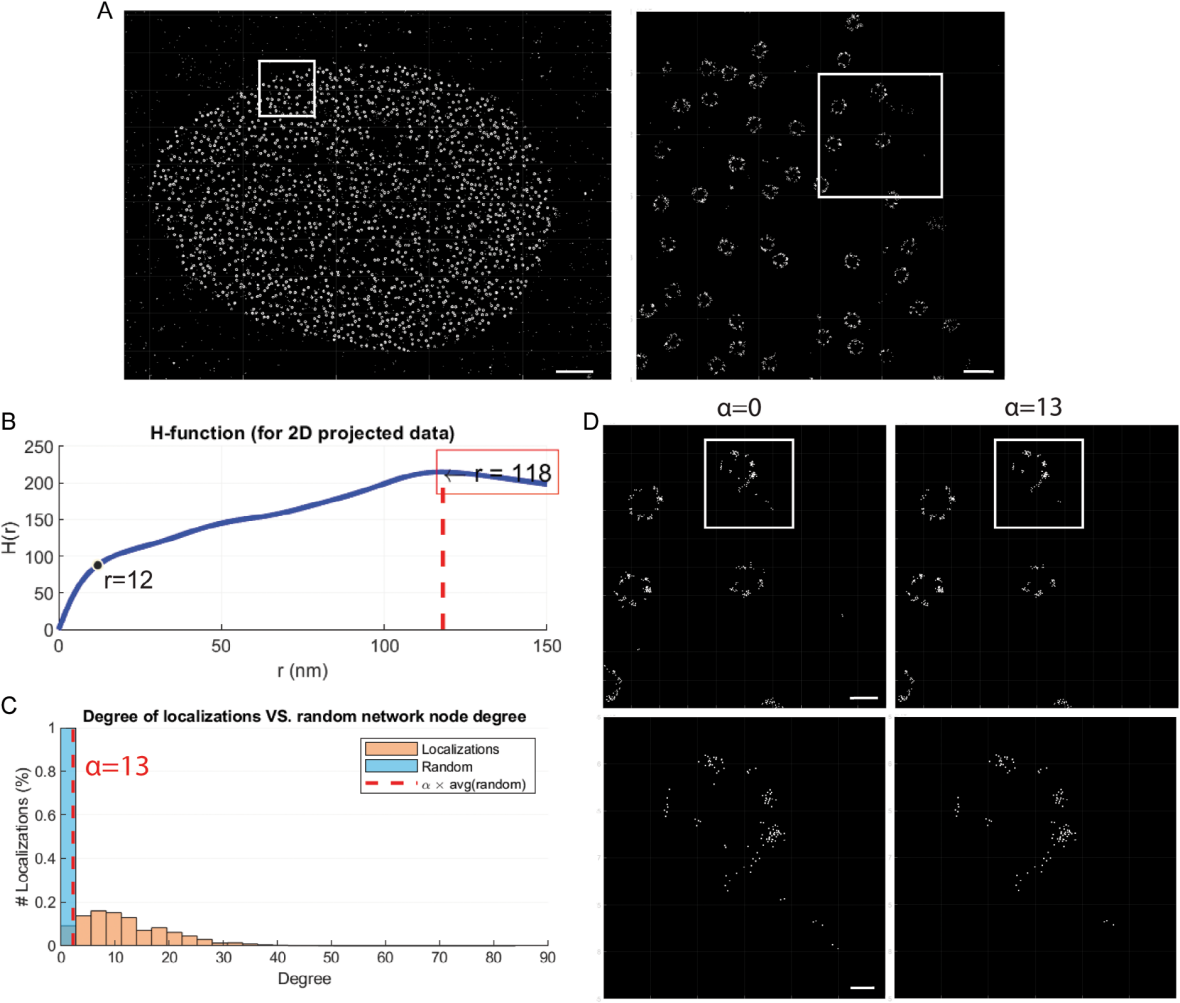
Preprocessing in SuperResNET using Merge & Network analysis and Filter tabs. A) 2D point cloud data for Nup96 acquired by dSTORM were loaded and visualized in SuperResNET software. Scale bar, 2 μm; zoom, 250 nm. B) Ripley's H‐function plotted against cluster radii. C) Degree distribution of random network (blue) and localization (orange). Dashed red line indicates degree value of *α* × average degree of random network. D) Effects of filtering at *α* = 0 and *α* = 13. Scale bar, 100 nm; zoom, 25 nm.

We applied SuperResNET to publicly available 2D dSTORM data for Nup96.^[^ ^20^ ^]^ The localization event list was loaded into SuperResNET GUI (**Figure** **2**A). Using the SuperResNET Merge & Network analysis tab, Ripley's H‐function was calculated for the loaded localizations. When plotting Ripley's H‐function H(r) against radius *r* (Figure 2B), a peak at *r* = 118 nm and an inflection point at *r* = 12 nm was identified. These correspond to the size of nuclear pores (the cluster scale) and Nup96 corners (modules or subcluster scale), respectively. To best filter the data to retain the corners, we generated a random network and plotted the degree distribution of that localization and that of the same data randomized at proximity threshold 12 nm (Figure 2C). Under the filter tab of SuperResNET (Figure 1), users can define filtering parameter α for denoising, removing localizations with degree lower than α×averagedegree of the random network. For the current dataset, *α* = 13 removed the random localizations (dashed red line in Figure 2C) with 7.8% of total localizations removed and considered not clustered at *r* = 12 nm scale. The effects of filtering are shown in Figure 2D. Localizations outside of nuclear pores were present at *α* = 0 and removed at *α* = 13, whereas the ring of nuclear pore was maintained.

**Figure 3:**
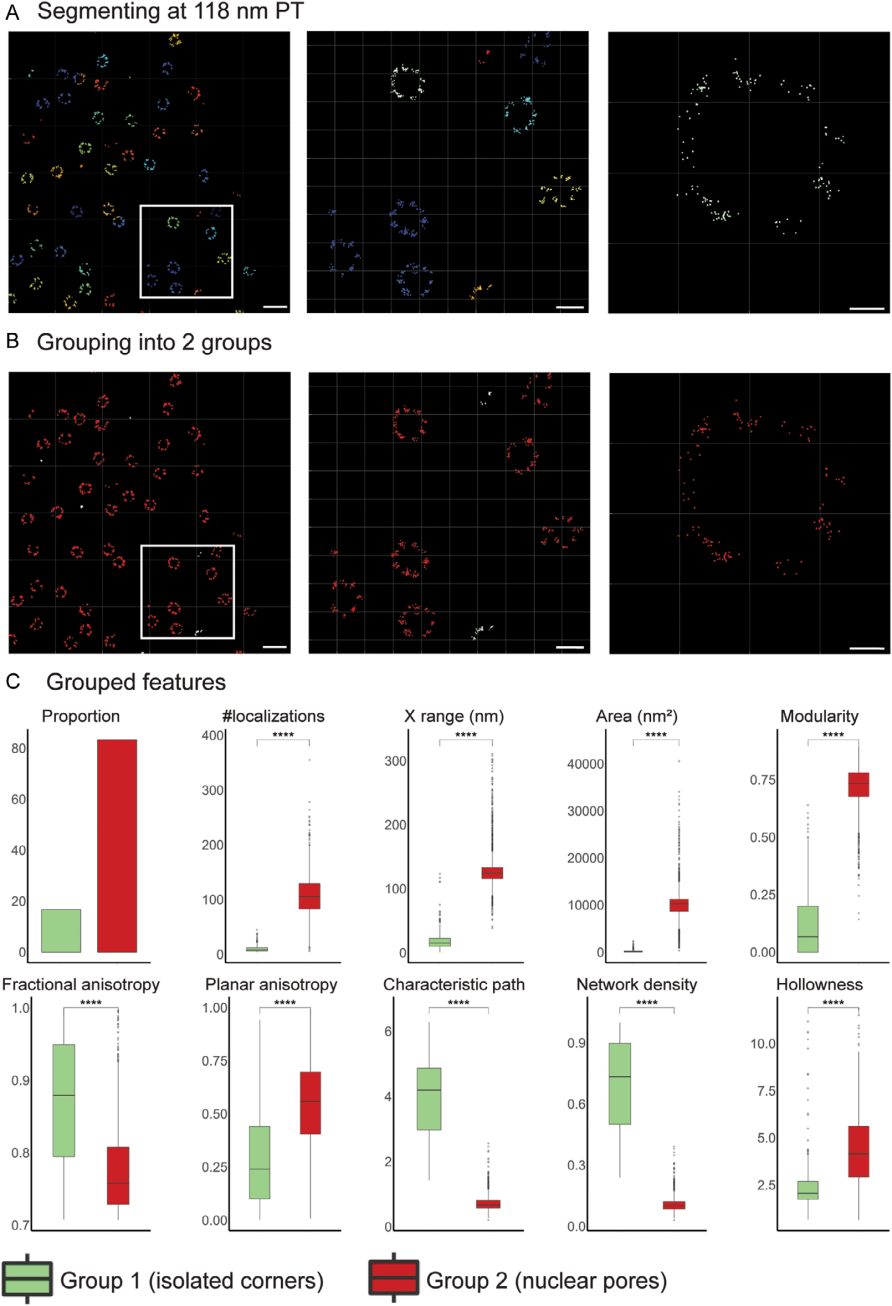
Segmentation and grouping of nuclear pores using segment and group tabs. A) Segmentation of nuclear pores using mean‐shift algorithm. B) Grouping of segmented blobs using blob features by K‐means clustering. Nuclear pores (Group 2, red) and isolated corners (Group 1, green) were classified into two separate groups. Scale bars, 250, 100, and 20 nm (left to right). C) Features of grouped blobs. Quantification of blob size, anisotropy (shape), and size features (*n* = 293 for Group 1 and *n* = 1469 for Group 2; two‐sided *t*‐test, ****p <  0.0001).

Based on the peak of Ripley's H‐function (*r* = 118 nm) (Figure 2B), nuclear pores were segmented using mean‐shift algorithm (**Figure** **3**A), and 30 features describing size, topology, hollowness, shape, and network were extracted from the segmented blobs. Formal definition of the used features is provided in the Methods, Supporting Information. Feature analysis of the segmented blobs showed that for area, average distance to centroid, characteristic path, modularity, network density, number of localizations, *x* range, and *y* range, there were two clear peaks (Figure S3, Supporting Information). We therefore identified 2 groups by K‐means clustering using the extracted features (Figure 3B), with 16.6% of blobs classified as Group 1 and 83.4% as Group 2; quantification of features after grouping is shown in Figure 3C. Group 1 blobs contain an average of 10 ± 0.4 localizations per blob, while Group 2 blobs contain 106.8 ± 1.0 localizations per blob. Group 1 blobs are smaller in size compared to Group 2, with *X* range of 19.9 ± 1.0 and 130.3 ± 0.9 nm, respectively. Group 1 blobs also show lower modularity and higher network density than Group 2, while Group 2 blobs are hollower than Group 1 (Figure 3C). This suggests that Group 1 blobs correspond to isolated clusters of Nup96 and Group 2 blobs correspond to more complete nuclear pores.

**Figure 4:**
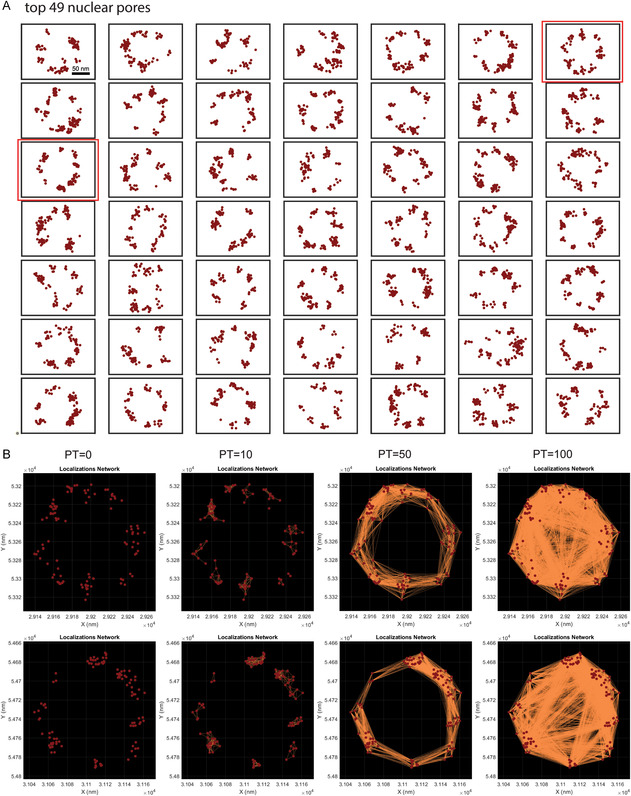
Network connection of representative nuclear pores retrieved using blob retrieval and network analysis tab. A) Localizations of top 49 representative nuclear pores. Boxed nuclear pores were shown in B) network connections at proximity threshold 0, 10, 50, and 100 nm.

The top 49 representative Group 2 blobs with features closest to the mean blob features were retrieved (**Figure** **4**A) using the SuperResNET blob retrieval tab (Figure 1). While most nuclear pores were incomplete and did not show 8 corners, some complete pores can be identified and network connection of two complete nuclear pores at different proximity thresholds (Figure 4B) was visualized using the individual blobs tab (Figure 1). Localizations within the same corners were connected into networks at proximity thresholds 10 nm, adjacent corners were connected at proximity threshold 50 nm, and corners on opposite sides of the nuclear pore were connected at proximity threshold 100 nm. These SuperResNET values correspond to the 42 nm distance between adjacent corners and 107 nm distance between opposing corners of the nucleopore complex, as determined by cryoelectron microscopy.^[^ ^21^ ^]^

### 2.3 SuperResNET Identified 2 Nup96 Molecules in Nup96 Corners by Modularity Analysis

**Figure 5:**
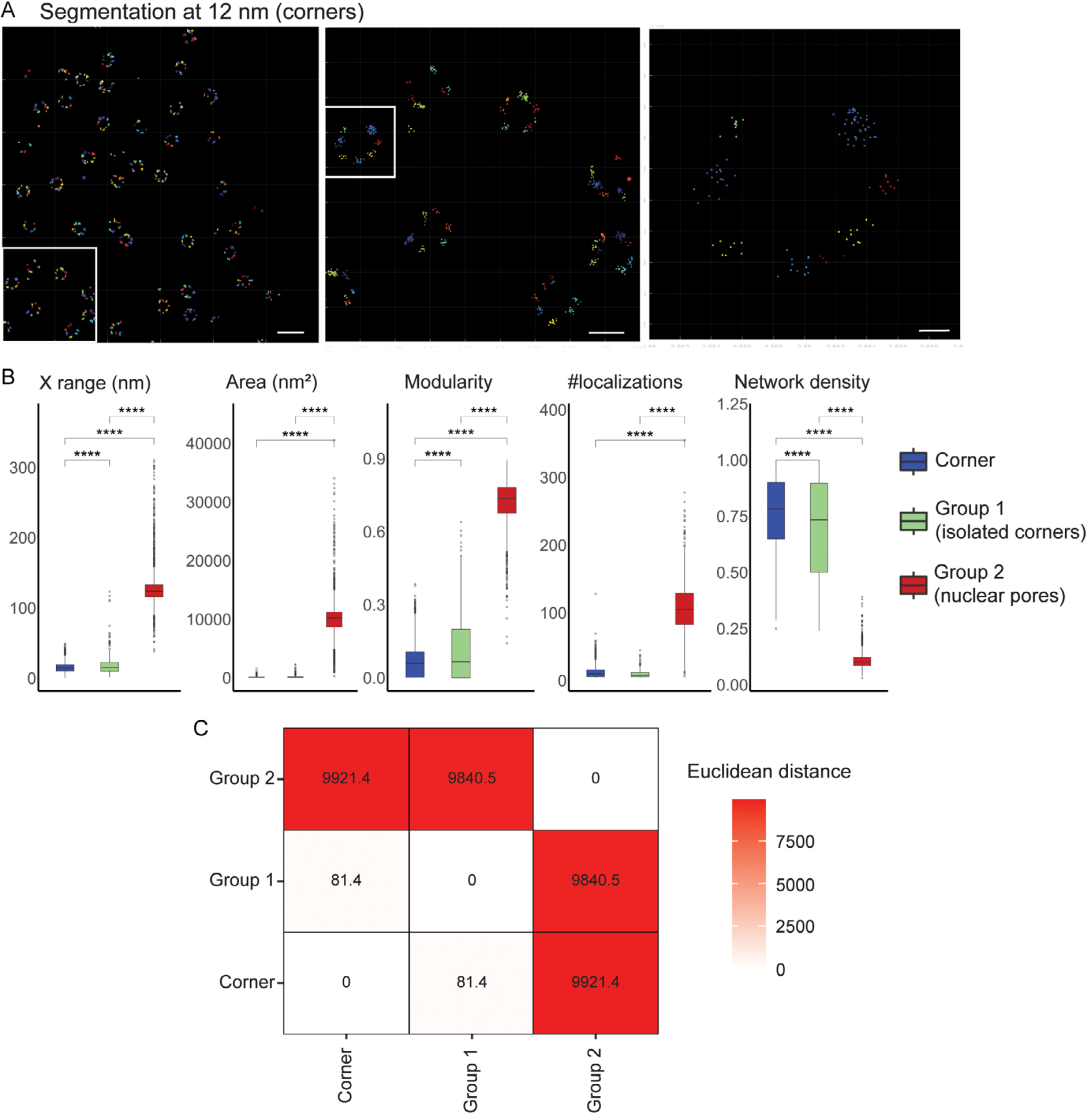
Segmentation of Nup96 corners using segment tab. A) Segmentation of nucleoporin 96 corners using the mean‐shift algorithm. Scale bars, 250, 100, and 20 nm (left to right). B) Features of segmented corners (blue), Group 1 (green) and Group 2 (red) in segmented pores (*n* = 12 636 for corner, *n* = 293 for Group 1 and *n* = 1469 for Group 2; ANOVA with Tukey post‐test, ****p < 0.0001). C) Euclidean distance of the group features of segmented corners and groups in segmented pores.

Based on the inflection point in Ripley's H‐function (Figure 2B), Nup96 corners were then segmented at a kernel bandwidth of 12 nm using mean‐shift algorithm (**Figure** **5**A). Corners contain 12 localizations on average and are 15.3 ± 0.1 nm in *X* range. They are low in modularity (0.06) and high in network density (0.77) (Figure 5B), which resemble the features of Group 1 blobs when segmenting at proximity threshold 118 nm. Euclidean distance was calculated using all features to compare segmented corners at proximity threshold 12 nm and previously identified Group 1 and Group 2 when segmented at proximity threshold 118 nm (Figure 5C). The Euclidean distance between segmented corners and Group 1 is highly similar and far from Group 2. This suggests that Group 1 blobs are isolated corners dissociated from complete nucleopore complexes.

**Figure 6:**
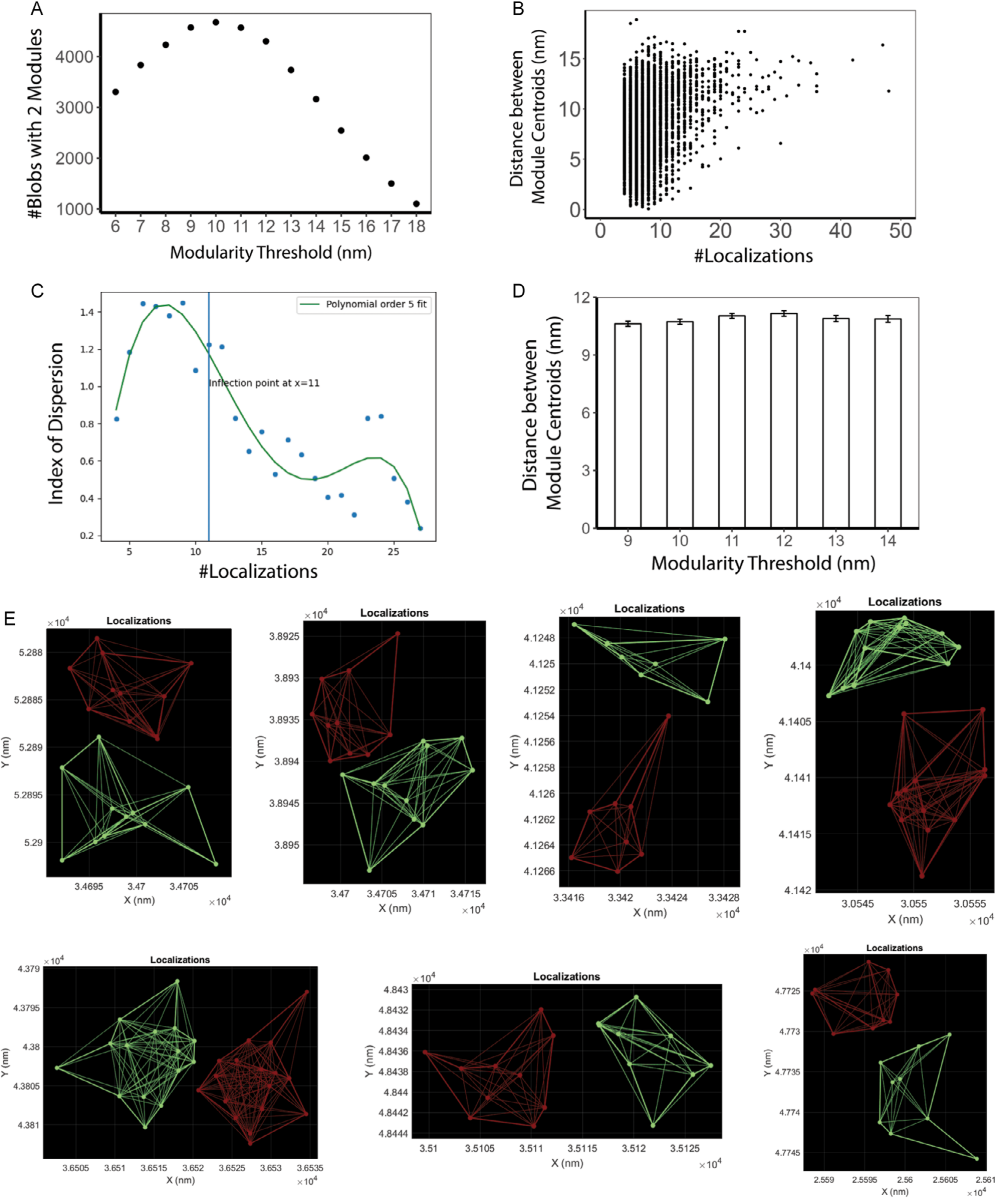
Modularity analysis of segmented Nup96 corners using blob modules tab. A) Relationship between modularity threshold and number of blobs with 2 modules. B) Relationship between number of localizations per blob and distance between centroid of 2 modules in Nup96 corners. Blobs with 2 modules were identified at proximity threshold 10 nm. C) Relationship between number of localizations and the index of dispersion of distance between module centroids. Green curve indicates polynomial order 5 fitting of data points, and blue line indicates inflection point of the curve. Blobs with 2 modules were identified at modularity threshold 10 nm. D) Mean distance between module centroids in blobs with greater than 11 localizations at modularity threshold 9–14 nm. E) Network connection of modules in representative blobs with 2 modules. See also Figure S4, Supporting Information for data with multiple modularity thresholds.

We then used the SuperResNET modularity analysis tab (Figure 1) to apply modularity analysis to the segmented corners, in which subnetworks of high‐density localizations are identified within segmented blobs.^[^ ^17^ ^]^ The number of modules per blob was identified at modularity threshold 6–20 nm and the number of blobs with 2 modules was plotted against the modularity threshold (**Figure** **6**A), which peaks at modularity threshold 10 nm. Distance between module centroids in blobs with 2 modules was measured, and those blobs containing fewer localizations show highly variable distance between module centroids (Figure 6B and Figure S4, Supporting Information), likely a consequence of poor identification of modules based on few localizations and low network density. To identify a threshold for blobs showing low variance of distance between modules, we calculated the index of dispersion (the ratio of variance to the mean) of distance between module centroids, plotted the value against number of localizations per blob, and identified the inflection point at 11 localizations using modularity thresholds from 9 to 14 nm (Figure 6C and Figure S4, Supporting Information). Blobs with fewer than 11 localizations were removed and mean distance between module centroids at modularity threshold 9–14 nm is shown in Figure 6D, showing similar mean distance between module centroids at different modularity thresholds, averaging 10.9 nm. Blobs with 2 modules at modularity threshold 10 nm have mean distance between module centroids of 10.7 ± 0.1 nm, corresponding to the 12 nm distance between Nup96 molecules measured by cryoelectron microscopy.^[^ ^21^ ^]^ Representative blobs with two modules were visualized using the blob modules tab (Figure 1) and shown in Figure 6E.

### 2.4 SuperResNET Effectively Segmented Nup107 Corners in 3D PAINT Data for Nup107

**Figure 7:**
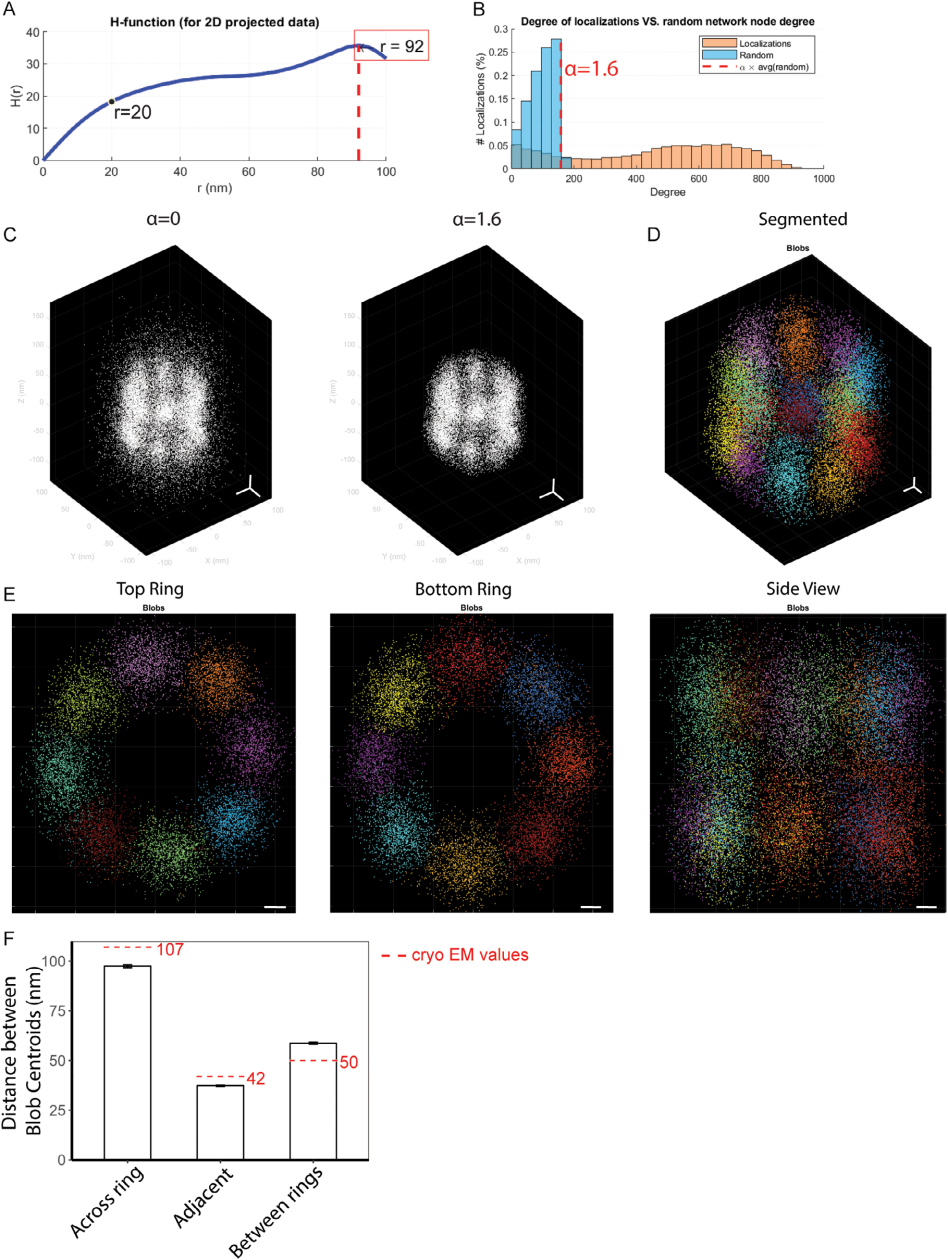
Preprocessing and segmentation of Nup107 corners in 3D SMLM data using SuperResNET. 3D point cloud data for Nup107 acquired by PAINT and reconstructed by fusion of 306 nuclear pores using joint registration of multiple point clouds method were obtained from Wang et al.^[^ ^22^ ^]^ A) Ripley's H‐function plotted against cluster radii. Inflection point of 20 nm was used as proximity threshold. B) Degree distribution of random network (blue) and localization (orange). Dashed red line indicates degree value of *α* × average degree of random network. C) 3D view of effects of filtering at *α* = 0 and *α* = 1.6. D) 3D view of segmented nucleoporin 107 corners using the mean‐shift algorithm. E) 2D view of each ring and side view. Scale bar, 10 nm. F) Mean distance between corner centroids. Dashed red lines indicate values measured by cryoelectron microscopy.^[^ ^21^ ^]^

We applied SuperResNET analysis to publicly available 3D reconstructed PAINT data for Nup107.^[^ ^22^ ^]^ Ripley's H‐function H(r) was calculated and plotted against radius *r* using Merge & Network analysis tab (**Figure** **7**A), where an inflection point at *r* = 20 nm was identified. We generated a random network and plotted the degree distribution of that localization and that of the same data randomized at proximity threshold 20 nm (Figure 7B), and used *α* = 1.6 (dashed red line in Figure 7B) to remove random localizations. The effects of filtering are shown in Figure 7C. Based on the inflection point of the Ripley's H‐function, Nup107 corners were segmented using mean‐shift algorithm with kernel bandwidth 20 nm and 16 corners arranged in 2 layers of eightfold symmetry were segmented (Figure 7D,E and Movie S1, Supporting Information). We then measured the distance between the centroid of segmented Nup107 corners across the ring, adjacent corners in the same ring, and corners in the two rings (Figure 7F), which were 97.4 ± 0.8, 37.4 ± 0.4, and 58.7 ± 0.5 nm, respectively, corresponding to the values measured by cryoelectron microscopy (dashed red lines in Figure 7F).^[^ ^21^ ^]^

## 3 Discussion

SuperResNET is a new GUI version of the previously described SMLM network analysis algorithm.^[^ ^15^, ^17^ ^]^ The algorithm represents a user‐friendly approach to filter, segment, group, and visualize the structures found within the point cloud of localizations generated by various SMLM approaches, including dSTORM. The GUI incorporates each element of the analysis pipeline into sequential tabs as well as novel computational modules including Ripley's H‐function, DBScan segmentation, feature normalization, t‐SNE and silhouette methods to assess grouping, pair‐wise feature visualization, and blob retrieval module. SuperResNET GUI provides a comprehensive and interactive environment for loading, preprocessing, postprocessing, and visualizing data. This integration streamlines the analysis process and eliminates the need for separate, noncohesive modules, a common limitation in previous tools. Here, we show that application of SuperResNET network analysis to publicly available dSTORM 2D Nup96 and 3D Nup107 data is able to reconstruct the eightfold symmetry of the nuclear pore as well as segment the nucleoporin corners. For the 2D Nup96 data, SuperResNET detected individual Nup96 molecules at ≈11 nm distance within the nucleopore corners, essentially attaining molecular resolution from dSTORM data. This analysis highlights the analytic improvements that network analysis brings to interpretation of SMLM data.

SuperResNET provides the ability to segment SMLM datasets at different proximity thresholds, essentially analyzing the extent to which localizations interact at varying distances. SuperResNET includes a Ripley's H‐function analysis tool that defines optimal clustering distances. For Nup96, SuperResNET provides the ability to segment and analyze complete nuclear pore complexes as well as the nucleoporin corners, based on the 118 and 12 nm kernel bandwidths obtained from Ripley's H‐function analysis. Euclidean comparison of feature of the two groups obtained at 118 nm and the corners obtained at 12 nm shows that the smaller Group 1 obtained at 118 nm proximity threshold closely matches the corners obtained at 12 nm proximity threshold. This suggests that Group 1 blobs represented isolated corners. These isolated corners are far less abundant than complete nuclear pores and are not seen as clusters larger than one corner. Whether they represent corners isolated from nuclear pore fragmentation due to fixation and processing of the samples or isolated corners within the nuclear membrane is not clear. Biological structures, from proteins to DNA to more complex protein complexes are modular in nature. SuperResNET provides the ability to compare the features of multiple structures within the cell and thereby define the relationship between various oligomeric structures derived from interaction between the same component molecule, in this case, Nup96.

Similarly, prior SuperResNET analysis of CAV1 defined caveolae and smaller scaffold structures and was able to show structural correspondence between the modules that comprise these different structures.^[^ ^17^ ^]^ This approach was incorporated into SuperResNET modularity analysis, in which submodules of larger structures are identified through increased proximity of subsets of localizations. Here, application of SuperResNET modularity analysis to the Nup96 corners was able to segregate clusters of localizations that were ≈12 nm apart corresponding to the two individual Nup96 proteins known to be found within nucleoporin corners. The ability to obtain molecular resolution at 12 nm from dSTORM highlights the potential of SuperResNET to define molecular architecture of cellular structures in situ within the cell. Indeed, application of SuperResNET to MINFLUX datasets of clathrin‐coated pits provides highly resolved clathrin pit and vesicle structures.^[^ ^23^ ^]^ The application of SuperResNET to these different structures highlights the versatility of this analysis approach which offers great potential for generalization. However, SuperResNET modularity analysis was unable to identify 2 modules in the segmented 3D Nup107 corners, likely a result of the extremely high number of localizations generated as a consequence of the particle averaging pipeline.^[^ ^22^ ^]^

Nevertheless, determination of molecular localizations from SMLM data remains challenging. A priori knowledge that nucleoporin corners comprise two Nup96 molecules^[^ ^21^ ^]^ led to a focus on two modules. Indeed, use of biological knowledge to inform interpretation of SMLM data is critical to guiding interpretation.^[^ ^24^ ^]^ Prior application of modularity analysis to CAV1 clusters was able to report on the number of modules in each structure based on local proximity of localizations.^[^ ^17^ ^]^ In that case, detected modules reported interaction between presumed CAV1 molecules as application of the SuperResNET merge module reduced local networks, formed due to the well‐characterized multiple blinking issue of SMLM.^[^ ^25^ ^]^ Multiple blinking occurs due to the fact that the same fluorophore can blink more than once; due to drift between blinking events, the multiple blinks do not necessarily coincide, forming a local cluster. Reduction of these local clusters is possible in SuperResNET using the variable threshold merge module. The merge threshold was previously set to 19–20 nm based on prior biological knowledge that ≈150 CAV1's formed a caveolae, and encompassed both localization precision of the detected blinks as well as error introduced by primary and secondary *F*(*ab*′)^2^ antibody labeling.^[^ ^16^ ^]^

As the SNAP‐tagged Nup96 data analyzed in the current study were already preprocessed using a temporal method by merging localizations that persist over consecutive frames,^[^ ^20^ ^]^ we did not merge localizations for this dataset. The fact that some individual Nup96 molecules within corners remained associated with multiple blinks within the localization precision of the acquisition of 13.6 nm^[^ ^20^ ^]^ indicates that preprocessing had not effectively removed multiple blinks. Further, as can be seen from the views of the most representative nucleopore complexes (Figure 4A), only few contain eight corners and the majority are incomplete. The combination of multiple blinking and incomplete coverage for this SMLM dataset of a highly defined structure, the nuclear pore complex, is an indication of the challenges faced when reporting on molecular structure with SMLM.^[^ ^20^ ^]^

SuperResNET's spatial approach to multiple blinking is best supported by saturation of labeling and blink acquisition. As can be seen in this study, in which localization merging in SuperResNET was not applied, these retained local clusters due to multiple blinking provide information that enabled modularity analysis of the corners and localization of individual Nup96 molecules. Importantly, a limited numbers of localizations for some corners resulted in poor identification of modules and highly variable reporting of distances between the two modules. Exclusion of corners with few localizations enabled accurate determination of the distance between the two clusters derived from the two Nup96 molecules within a nucleopore corner, a distance that was below the localization precision of the acquisition. This highlights the power of cluster analysis for SMLM data and also the way the multiple blinking “problem” of SMLM can be leveraged to inform on underlying molecular structure.

One approach to correct for incomplete labeling is particle averaging, where multiple identical copies of the structure of interest are combined to reconstruct a “super‐particle” with high labeling density and signal‐to‐noise ratio. However, common template‐based methods^[^ ^26^, ^27^ ^]^ are prone to template bias, and methods derived from single particle analysis for cryoelectron microscopy^[^ ^28^, ^29^ ^]^ are not compatible with 3D SMLM data. Recent development of template‐free 3D particle fusion methods^[^ ^22^, ^30^ ^]^ overcomes these limitations, but assumes a single consistent structure and is therefore unable to report on structural variation within the dataset. In contrast, with extended acquisition of blinks and more coverage of the imaged structures, SuperResNET provides the user with flexibility to report on structural diversity in situ within the cell without model‐fitting, providing opportunities for biological discovery.

## 4 Experimental Section

4.1

4.1.1

##### Data Source

List of localizations of publicly available 2D SMLM data for Nup96 was obtained from Thevathasan et al.^[^ ^20^ ^]^ To summarize, U20S cells expressing Nup96‐SNAP labeled with Alexa Fluor 647‐benzylguanine were fixed and imaged using dSTORM. Localizations generated by custom software written in MATLAB were corrected for drift, merged to remove localizations persistent over consecutive frames, and filtered by localization precision and fitting quality.^[^ ^20^ ^]^

The localization list of publicly available 3D PAINT data for Nup107 was obtained from Wang et al.^[^ ^22^ ^]^ The data were reconstructed from 306 Nup107 particles acquired by 3D astigmatic PAINT processed using particle averaging pipeline based on the Joint Registration of Multiple Point Clouds method and Gaussian Mixtures Model.^[^ ^22^ ^]^

##### SuperResNET GUI

The SuperResNET GUI was built in MATLAB and based on previous SMLM network analysis software^[^ ^15^ ^]^ and includes addition of novel computational modules for Ripley's H‐function, DBScan segmentation method, K‐means clustering and feature normalization, pairwise feature visualization, and blob retrieval. Here, SuperResNET GUI was used to analyze the publicly available 2D dSTORM Nup96 data referenced above. Proximity thresholds for the analysis were determined using Ripley's H‐function, a normalized Ripley's K‐function whose expected value is 0. To remove background and random localization, we constructed a random localization network and extracted node degree, a measure of network connectivity, of each localization at proximity threshold 12 nm, compared the distribution of random network/graph with experimental data and retained localizations with degree greater than the average network degree of the random graph multiplied by a scalar alpha (*α*). For the 3D PAINT Nup107 dataset, a proximity threshold of 20 nm was used based on Ripley's H‐function.

The mean‐shift algorithm, a density‐based clustering method,^[^ ^31^ ^]^ was used to segment nuclear pores and Nup96 corners in the denoised localizations using kernel bandwidth determined from Ripley's H‐function, and 30 features of each blob (Figure S3, Supporting Information) were extracted to describe the size, shape/anisotropy, topology, and network features and corresponding statistical features (minimum, maximum, mean/average, median, and standard deviation). The 30 features were then used to characterize blob groups using K‐means clustering, where 2 groups were identified in segmented nuclear pores and 1 in segmented corners. For the 3D PAINT Nup107 dataset, Nup107 corners in the denoised localizations were segmented, and 1 group was identified.

Modularity analysis that identifies modules (communities) with high network connectivity using the Newman method has been described previously^[^ ^17^ ^]^ and was applied to segmented 2D Nup96 corners at modularity proximity threshold 6–18 nm. Corners with 2 modules were identified and distance between the module centroids was measured.

Additional details on SuperResNET capabilities can be found in the Methods, Supporting Information. SuperResNET GUI is available as Software, Supporting Information (Instructions for reviewers to download SuperResNET GUI can be found in the Supporting Information “SuperResNET software installation guide.docx”.). Updated versions of the software can also be found at https://www.medicalimageanalysis.com/software/superresnet. SuperResNET GUI can be applied to a single uploaded SMLM dataset. Parallel analysis of multiple datasets is available through the previously described batch analysis version of SuperResNET^[^ ^15^, ^16^ ^]^ can be accessed at https://github.com/NanoscopyAI. SuperResNET GUI is compiled for Windows and Mac operating systems. The software manual shows the installation process and provides documentation on how to use the software to analyze biological SMLM data. The software comes with a license for academic use only.

## Conflict of Interest

The authors declare no conflict of interest.

## Author Contributions

**Yahongyang Lydia Li** and **Ismail M. Khater** should be considered joint first author. **Ivan R. Nabi** and **Ghassan Hamarneh** should be considered joint senior author. **Yahongyang Lydia Li**: conceptualization (equal); data curation (lead); formal analysis (lead); investigation (equal); methodology (supporting); writing—original draft (lead); writing—review and editing (equal). **Ismail M. Khater**: conceptualization (equal); investigation (equal); methodology (equal); software (lead). **Christian Hallgrimson**: data curation (supporting); formal analysis (supporting); methodology (supporting); software (supporting). **Ben Cardoen**: formal analysis (supporting). **Timothy H. Wong**: software (supporting). **Ghassan Hamarneh**: conceptualization (supporting); investigation (supporting); project administration (supporting); software (supporting); supervision (equal); writing—review and editing (supporting). **Ivan R. Nabi**: conceptualization (equal); funding acquisition (lead); investigation (equal); project administration (equal); resources (lead); supervision (equal); writing—review and editing (equal). **Y. Lydia Li, Ismail M. Khater**, **Ghassan Hamarneh**, and **Ivan R. Nabi** have contributed equally to this work.

## Supporting information

Supplementary Material

## Data Availability

The data that support the findings of this study are openly available in BioImage Archive at https://www.ebi.ac.uk/biostudies/BioImages/studies/S‐BIAD8, reference number 181214. These data were derived from the following resources available in the public domain: https://www.ebi.ac.uk/biostudies/BioImages/studies/S‐BIAD8; https://github.com/wexw/Joint‐Registration‐of‐Multiple‐Point‐Clouds‐for‐Fast‐Particle‐Fusion‐in‐Localization‐Microscopy; …;.

## References

[1] E. Betzig , G. H. Patterson , R. Sougrat , O. W. Lindwasser , S. Olenych , J. S. Bonifacino , M. W. Davidson , J. Lippincott‐Schwartz , H. F. Hess , Science 2006, 313, 1642. 16902090 10.1126/science.1127344

[2] S. T. Hess , T. P. Girirajan , M. D. Mason , Biophys. J. 2006, 91, 4258. 16980368 10.1529/biophysj.106.091116 PMC1635685

[3] M. Heilemann , S. van de Linde , M. Schuttpelz , R. Kasper , B. Seefeldt , A. Mukherjee , P. Tinnefeld , M. Sauer , Angew. Chem. Int. Ed. Engl. 2008, 47, 6172. 18646237 10.1002/anie.200802376

[4] M. J. Rust , M. Bates , X. Zhuang , Nat. Methods 2006, 3, 793. 16896339 10.1038/nmeth929 PMC2700296

[5] R. Jungmann , M. S. Avendano , J. B. Woehrstein , M. Dai , W. M. Shih , P. Yin , Nat. Methods 2014, 11, 313. 24487583 10.1038/nmeth.2835 PMC4153392

[6] J. Schnitzbauer , M. T. Strauss , T. Schlichthaerle , F. Schueder , R. Jungmann , Nat. Protoc. 2017, 12, 1198. 28518172 10.1038/nprot.2017.024

[7] F. Balzarotti , Y. Eilers , K. C. Gwosch , A. H. Gynna , V. Westphal , F. D. Stefani , J. Elf , S. W. Hell , Science 2017, 355, 606. 28008086 10.1126/science.aak9913

[8] K. C. Gwosch , J. K. Pape , F. Balzarotti , P. Hoess , J. Ellenberg , J. Ries , S. W. Hell , Nat. Methods 2020, 17, 217. 31932776 10.1038/s41592-019-0688-0

[9] S. C. M. Reinhardt , L. A. Masullo , I. Baudrexel , P. R. Steen , R. Kowalewski , A. S. Eklund , S. Strauss , E. M. Unterauer , T. Schlichthaerle , M. T. Strauss , C. Klein , R. Jungmann , Nature 2023, 617, 711. 37225882 10.1038/s41586-023-05925-9 PMC10208979

[10] B. Huang , H. Babcock , X. Zhuang , Cell 2010, 143, 1047. 21168201 10.1016/j.cell.2010.12.002 PMC3272504

[11] S. Liu , P. Hoess , J. Ries , Annu. Rev. Biophys. 2022, 51, 301. 35119945 10.1146/annurev-biophys-102521-112912

[12] D. Sage , T. A. Pham , H. Babcock , T. Lukes , T. Pengo , J. Chao , R. Velmurugan , A. Herbert , A. Agrawal , S. Colabrese , A. Wheeler , A. Archetti , B. Rieger , R. Ober , G. M. Hagen , J. B. Sibarita , J. Ries , R. Henriques , M. Unser , S. Holden , Nat. Methods 2019, 16, 387. 30962624 10.1038/s41592-019-0364-4 PMC6684258

[13] Y. Hyun , D. Kim , Comput. Struct. Biotechnol. J. 2023, 21, 879. 36698968 10.1016/j.csbj.2023.01.006 PMC9860261

[14] I. M. Khater , I. R. Nabi , G. Hamarneh , Patterns 2020, 1, 100038. 33205106 10.1016/j.patter.2020.100038 PMC7660399

[15] I. M. Khater , F. Meng , T. H. Wong , I. R. Nabi , G. Hamarneh , Sci. Rep. 2018, 8, 9009. 29899348 10.1038/s41598-018-27216-4 PMC5998020

[16] I. M. Khater , S. T. Aroca‐Ouellette , F. Meng , I. R. Nabi , G. Hamarneh , PLoS One 2019, 14, e0211659. 31449531 10.1371/journal.pone.0211659 PMC6709882

[17] I. M. Khater , Q. Liu , K. C. Chou , G. Hamarneh , I. R. Nabi , Sci. Rep. 2019, 9, 9888. 31285524 10.1038/s41598-019-46174-z PMC6614455

[18] I. M. Khater , F. Meng , I. R. Nabi , G. Hamarneh , Bioinformatics 2019, 35, 3468. 30759191 10.1093/bioinformatics/btz113 PMC6748737

[19] T. H. Wong , I. M. Khater , B. Joshi , M. Shahsavari , G. Hamarneh , I. R. Nabi , Sci. Rep. 2021, 11, 7810. 33833286 10.1038/s41598-021-86770-6 PMC8032680

[20] J. V. Thevathasan , M. Kahnwald , K. Cieslinski , P. Hoess , S. K. Peneti , M. Reitberger , D. Heid , K. C. Kasuba , S. J. Hoerner , Y. Li , Y. L. Wu , M. Mund , U. Matti , P. M. Pereira , R. Henriques , B. Nijmeijer , M. Kueblbeck , V. J. Sabinina , J. Ellenberg , J. Ries , Nat. Methods 2019, 16, 1045. 31562488 10.1038/s41592-019-0574-9 PMC6768092

[21] A. von Appen , J. Kosinski , L. Sparks , A. Ori , A. L. DiGuilio , B. Vollmer , M. T. Mackmull , N. Banterle , L. Parca , P. Kastritis , K. Buczak , S. Mosalaganti , W. Hagen , A. Andres‐Pons , E. A. Lemke , P. Bork , W. Antonin , J. S. Glavy , K. H. Bui , M. Beck , Nature 2015, 526, 140. 26416747 10.1038/nature15381 PMC4886846

[22] W. Wang , H. Heydarian , T. Huijben , S. Stallinga , B. Rieger , Bioinformatics 2022, 38, 3281. 35552632 10.1093/bioinformatics/btac320 PMC9191212

[23] T. H. Wong , I. M. Khater , C. Hallgrimson , Y. L. Li , G. Hamarneh , I. R. Nabi , *biorxiv:2024.03.07.583946* 2024.

[24] I. R. Nabi , B. Cardoen , I. M. Khater , G. Gao , T. H. Wong , G. Hamarneh , *arXiv:2305.17193*HYPERLINK 2023.

[25] L. G. Jensen , T. Y. Hoh , D. J. Williamson , J. Griffie , D. Sage , P. Rubin‐Delanchy , D. M. Owen , Nat. Methods 2022, 19, 594. 35545712 10.1038/s41592-022-01463-w

[26] A. Loschberger , S. van de Linde , M. C. Dabauvalle , B. Rieger , M. Heilemann , G. Krohne , M. Sauer , J. Cell Sci. 2012, 125, 570. 22389396 10.1242/jcs.098822

[27] J. Broeken , H. Johnson , D. S. Lidke , S. Liu , R. P. Nieuwenhuizen , S. Stallinga , K. A. Lidke , B. Rieger , Methods Appl. Fluoresc. 2015, 3, 014003. 25866640 10.1088/2050-6120/3/1/014003 PMC4390137

[28] D. Salas , A. Le Gall , J. B. Fiche , A. Valeri , Y. Ke , P. Bron , G. Bellot , M. Nollmann , Proc. Natl. Acad. Sci. U.S.A. 2017, 114, 9273. 28811371 10.1073/pnas.1704908114 PMC5584428

[29] C. Sieben , N. Banterle , K. M. Douglass , P. Gonczy , S. Manley , Nat. Methods 2018, 15, 777. 30275574 10.1038/s41592-018-0140-x PMC6173288

[30] H. Heydarian , M. Joosten , A. Przybylski , F. Schueder , R. Jungmann , B. V. Werkhoven , J. Keller‐Findeisen , J. Ries , S. Stallinga , M. Bates , B. Rieger , Nat. Commun. 2021, 12, 2847. 33990554 10.1038/s41467-021-22006-5 PMC8121824

[31] D. Comaniciu , P. Meer , IEEE Trans. Pattern Anal. 2002, 24, 603.

[32] L. Pelkmans , M. Zerial , Nature 2005, 436, 128. 16001074 10.1038/nature03866

